# GL-1196 Suppresses the Proliferation and Invasion of Gastric Cancer Cells via Targeting PAK4 and Inhibiting PAK4-Mediated Signaling Pathways

**DOI:** 10.3390/ijms17040470

**Published:** 2016-04-11

**Authors:** Jian Zhang, Hong-Yan Zhang, Jian Wang, Liang-Hao You, Rui-Zhi Zhou, Dong-Mei Zhao, Mao-Sheng Cheng, Feng Li

**Affiliations:** 1 Department of Cell Biology, Key Laboratory of Cell Biology, Ministry of Public Health and Key Laboratory of Medical Cell Biology, Ministry of Education, China Medical University, Shenyang 110122, China; jiancmu@163.com (J.Z.); zhanghongyan516@163.com (H.-Y.Z.); 18640209060@163.com (L.-H.Y.); zrzym111@126.com (R.-Z.Z.); 2 Key Laboratory of Structure-Based Drug Design & Discovery of Ministry of Education, Shenyang Pharmaceutical University, Shenyang 110016, China; jianwang.chem@gmail.com (J.W.); dongmeiz-67@163.com (D.-M.Z.); mscheng@263.net (M.-S.C.)

**Keywords:** GL-1196, PAK4, small molecular compound, gastric cancer

## Abstract

Gastric cancer, which is the most common malignant gastrointestinal tumor, has jumped to the third leading cause of cancer-related mortality worldwide. It is of great importance to identify novel and potent drugs for gastric cancer treatment. P21-activated kinase 4 (PAK4) has emerged as an attractive target for the development of anticancer drugs in consideration of its vital functions in tumorigenesis and progression. In this paper, we reported that GL-1196, as a small molecular compound, effectively suppressed the proliferation of human gastric cancer cells through downregulation of PAK4/c-Src/EGFR/cyclinD1 pathway and CDK4/6 expression. Moreover, GL-1196 prominently inhibited the invasion of human gastric cancer cells in parallel with blockage of the PAK4/LIMK1/cofilin pathway. Interestingly, GL-1196 also inhibited the formation of filopodia and induced cell elongation in SGC7901 and BGC823 cells. Taken together, these results provided novel insights into the potential therapeutic strategy for gastric cancer.

## 1. Introduction

Gastric cancer is one of the most common malignant tumors in the world. It is recently reported that the worldwide mortality of gastric cancer is the third leading cause of cancer related deaths with an estimated 723,000 deaths in 2012 [1]. Despite advances in treatment approaches being conducted in recent decades, the outcomes of patients with advanced gastric cancer are still unsatisfactory [2]. Thus, to investigate novel therapeutic targets and identify effective drugs to improve the survival of patients with gastric cancer become very important.

A large number of studies have shown that the dysfunction of signaling pathways in cancer cells is the major reason that tumorigenesis and metastasis occur. As early as the end of 2004, Nature Reviews Cancer and Nature Reviews Drug Discovery discussed the important role of the protein kinase signaling pathway in tumors and identified protein kinase as a target for cancer therapy [3,4]. The p21-activated kinases (PAKs), a family of serine/threonine protein kinases, act as effectors of Rac and Cdc42 [5]. The six PAK family members are classified into group I (PAK1-3) and group II (PAK4-6) [6]. PAK4 was the first member of Group II PAKs to be identified and was the most extensively and profoundly studied. It has been shown to be overexpressed or genetically amplified in a variety of cancer cell lines and tumor types including breast, prostate, pancreas and stomach [7,8,9]. More importantly, PAK4 was recently reported to be overexpressed in metastatic gastric cancer patients [10], and the high level of activated PAK4 correlates with poor prognosis [11,12].

PAK4 was confirmed to play an important role in many fundamental cellular processes. Expression of wild-type and constitutively active PAK4 induced a significant increase in cellular proliferation in ovarian cancer cells [13], and it was reported to repress TGF-β-mediated growth inhibition in gastric cancer cells [14]. Subsequent studies demonstrated that PAK4 could regulate cell proliferation involving the c-Src/EGFR/cyclinD1 pathway, as well as regulating the expression of CDC25A and CDK6 [13,15,16]. On the other hand, as a known cytoskeletal regulatory kinase, PAK4 could interact with microtubules via binding with Cdc42 and GEF-H1, which subsequently impact cellular morphology and motility [17,18]. It also has been shown to promote cell migration and invasion through HGF/LIMK1/cofilin pathway [19], as well as interacting with DGCR6L and phosphorylating SCG10 [9,20]. Moreover, PAK4 could mediate the induction of filopodia in response to activated Cdc42 [17], inhibit cell adhesion [21] and promote anchorage-independent growth [7,21,22]. Many of these functions rely on the kinase activity of PAK4.

To explore potential drug candidates against gastric cancer, we have screened a series of small molecular compounds from Specs that provides compounds that are required in drug discovery for their inhibition efficacy towards gastric cancer cells. Fortunately, we found that GL-1196 could effectively suppress the proliferation and invasion of gastric cancer cells. More importantly, we identify this novel small molecular compound suppressing human gastric cancer cells via targeting PAK4, and provide a potential therapeutic strategy for gastric cancer by blockade of PAK4 signaling.

## 2. Results

### 2.1. GL-1196 Suppresses the Proliferation of Gastric Cancer Cells and Mediates Cell Cycle Arrest at G1 Phase

**Figure 1:**
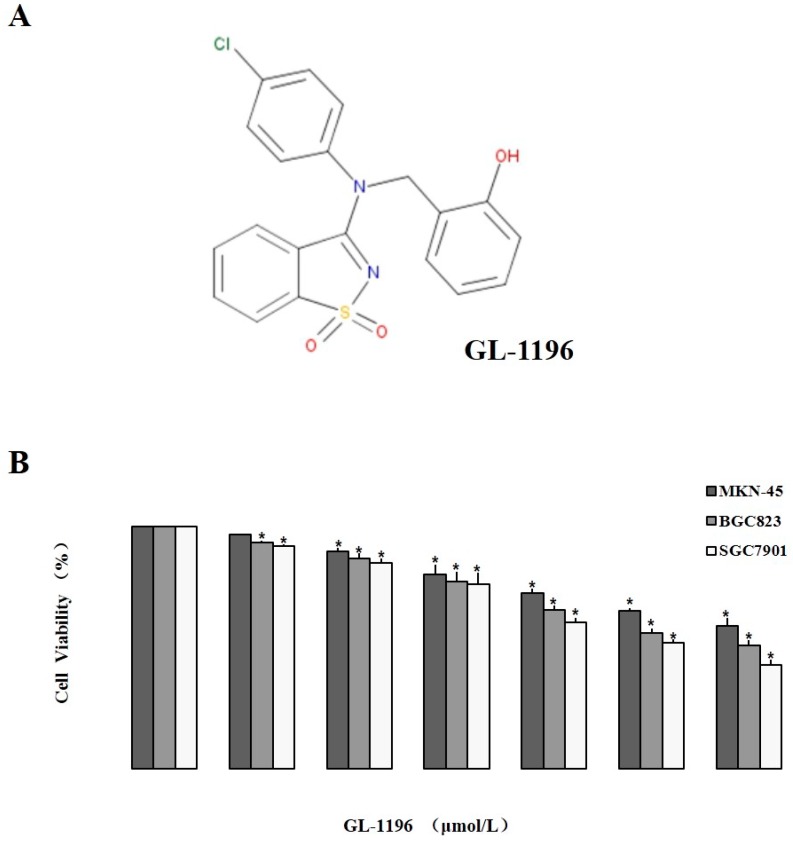
Chemical structure and the inhibitory proliferative effect on human gastric cancer cells of GL-1196. (**A**) chemical structure of GL-1196 (C_20_H_15_ClN_2_O_3_S); (**B**) the effect of GL-1196 on proliferation of human gastric cancer cells by MTT assay. The cell viability is shown as bar diagram ± SEM, * *p* < 0.05.

**Figure 2:**
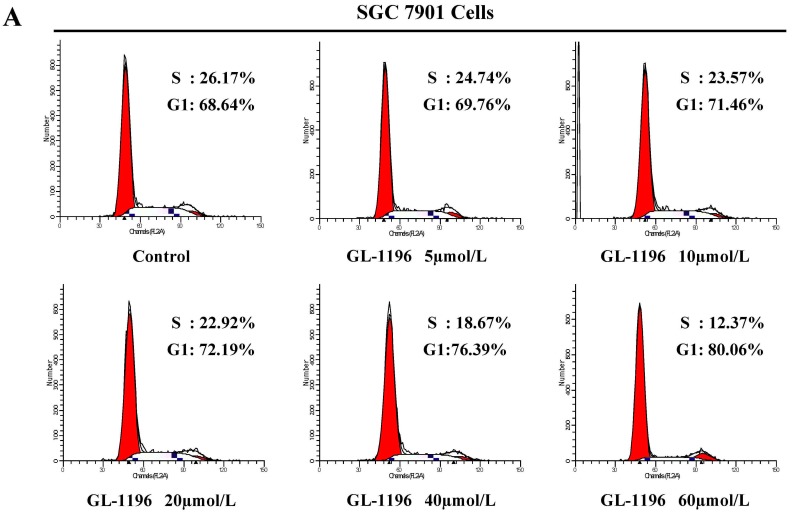

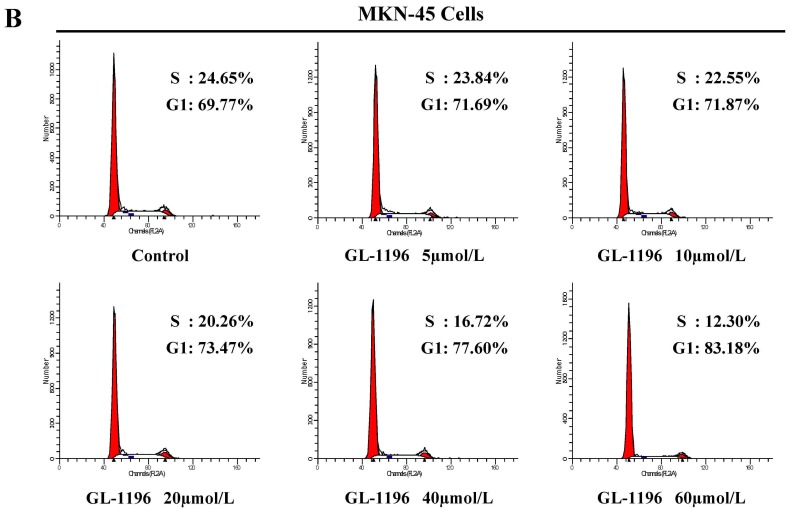
GL-1196 suppresses the transition of SGC7901 (**A**) and MKN-45 (**B**) cells from G1 to S phase.

We used MKN-45, BGC823 and SGC7901 cells to detect the effect of GL-1196 on the proliferation of these human gastric cancer cells. Just as the results shown in MTT assay, GL-1196 treatment decreased the proliferation of MKN-45, BGC823 and SGC7901 cells in a dose-dependent manner (Figure 1B). To further investigate the mechanism underlying GL-1196 that inhibited the growth of gastric cancer cells, two cell lines involving SGC7901 and MKN-45 were exposed to different concentrations of GL-1196 for 24 h, and then cell cycle analyses were performed. The results showed that GL-1196 effectively induced a concentration-dependent increase in the percentage of cells in G1 phase and decrease in S phase compared with control, indicating that GL-1196 could arrest SGC7901 and MKN-45 cells at the G1 phase of the cell cycle (Figure 2).

### 2.2. GL-1196 Represses the Invasive Potential of Gastric Cancer Cells

**Figure 3:**
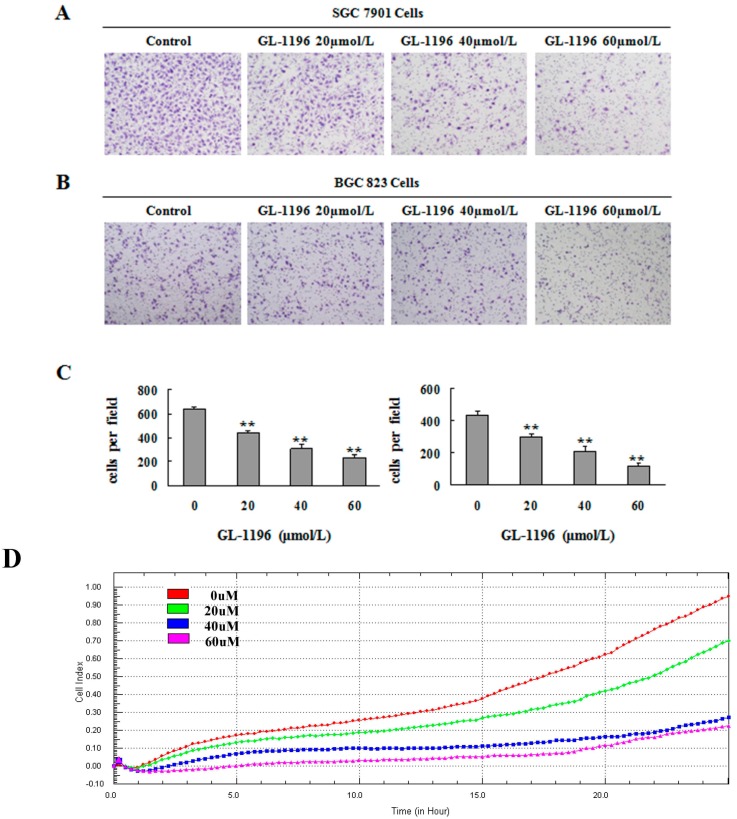
GL-1196 suppresses the invasive capacity of human gastric cancer cells. The invasive capability of SGC7901 (**A**) and BGC823 (**B**) cells was evaluated by chemotaxis chamber matrigel invasion assay. The magnification is 100×, and the number of invading cells is shown as bar diagram ± SEM; (**C**) the left one is for SGC7901 cells; the right one is for BGC823 cells, ** *p* < 0.01; (**D**) the effect of GL-1196 on MKN-45 cells invasive ability was detected by real time invasion monitoring.

The effect of GL-1196 on invasion of SGC7901 and BGC823 cells were analyzed by transwell assay. Results showed that GL-1196 potently decreased the invasion of these two gastric cancer cell lines in a dose-dependent manner (Figure 3A,B). Furthermore, we also detected the inhibitory effect on invasion of GL-1196 by real-time invasion monitoring. As the data collected from the xCELLigence system showed, a dose-dependent decrease in cell invasiveness was seen following treatment with GL-1196 in MKN-45 cells (Figure 3D).

### 2.3. GL-1196 Inhibits PAK4 Kinase Activity

**Figure 4:**
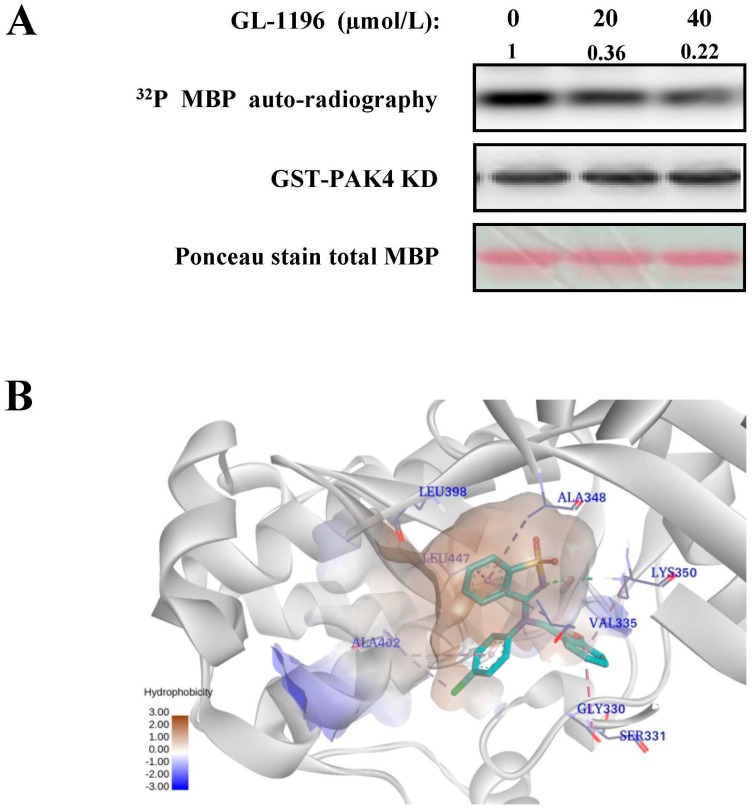
GL-1196 inhibits PAK4 kinase activity. (**A**) the effect of GL-1196 on PAK4 kinase activity was detected by kinase assay; (**B**) the binding mode of GL-1196 within PAK4 binding site. The green structure indicates the chemical structure of GL-1196.

It is reported that PAK4 participates in the regulation of proliferation, invasion and morphology in multiple cancer cells. In addition, many of these functions rely on its kinase activity. Thus, we applied kinase assay to detect if GL-1196 had the inhibitory potency on PAK4 and the results indicated that GL-1196 could markedly inhibit the PAK4 kinase activity in a dose-dependent manner (Figure 4A). In addition, to detect the modes at which GL-1196 interact with PAK4, the docking simulations were performed using Glide in Schrödinger version 2014. As shown in Figure 4B, the compound GL-1196 forms a conventional H-bonding interaction and seven π-alkyl interactions with receptor PAK4.

### 2.4. GL-1196 Suppresses the Invasive Capability of Gastric Cancer Cells via Targeting PAK4

**Figure 5:**
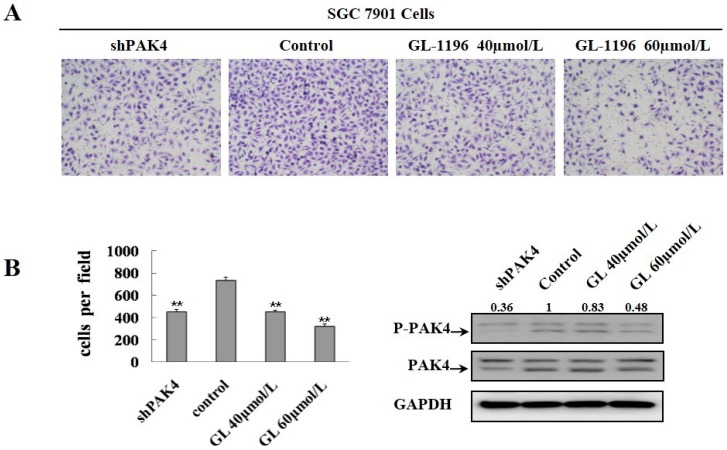
GL-1196 treatment showed the similar inhibitory effect on cell invasion by PAK4 knockdown. (**A**) the invasive ability of SGC7901 treated with GL-1196 and in which PAK4 knockdown was evaluated by chemotaxis chamber matrigel invasion assay. The magnification is 100×, and the number of invading cells is shown as bar diagram ± SEM (**B** left), ** *p* < 0.01. Western blot analysis shows the protein level of PAK4 in cells (**B** right).

**Figure 6:**
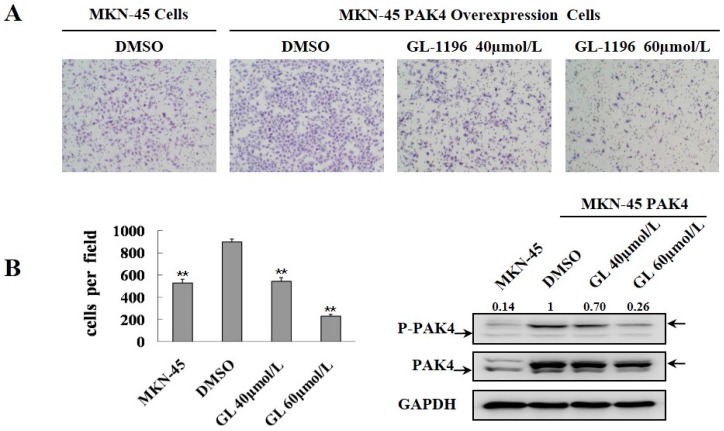
GL-1196 treatment exhibited the same inhibitory invasive effect on PAK4-overexpression MKN-45 cells as the control MKN-45 cells. (**A**) the invasive ability of PAK4-overexpression and control MKN-45 cells was evaluated by using a Boyden chamber matrigel invasion assay. The magnification is 100×, and the number of cells invading is shown as bar diagram ± SEM (**B** left), ** *p* < 0.01. Western blot analysis shows the protein level of PAK4 in cells (**B** right).

PAK4 activity promotes cell invasiveness, and moreover, GL-1196 inhibits the kinase activity of PAK4; thus, we detected if the inhibitory effect of GL-1196 on cell invasion was due to its impact on PAK4 kinase activity. Then, transwell assays were conducted to compare the invasive capability between the SGC7901 cells treated with GL-1196 and PAK4 knockdown. As expected, the results revealed that GL-1196 treatment showed the similar inhibitory effect on cell invasion to the impact of PAK4 knockdown (Figure 5). Furthermore, GL-1196 treatment exhibited the same inhibitory invasive effect on PAK4-overexpression MKN-45 cells that were infected with lentivirus carrying PAK4 as the MKN-45 cells infected with lentivirus carrying vector (Figure 6).

### 2.5. GL-1196 Inhibits the Filopodia Formation of Gastric Cancer Cells

**Figure 7:**
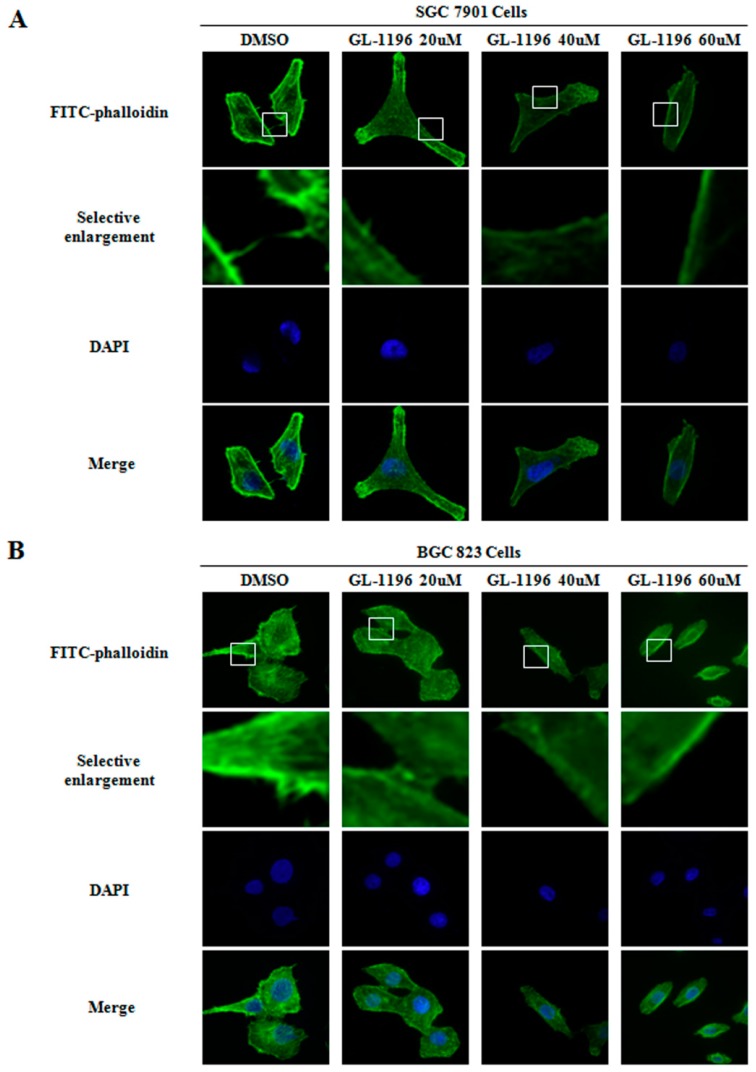
GL-1196 inhibits the formation of filopodia in SGC7901 (**A**) and BGC823 (**B**) cells. The magnification of the images is 400×. The blue fluorescence shows the nucleus stained with DAPI. Selective enlargement represents the magnifying images of scope within the white squares.

As is well known, filopodia are actin-containing spikes that could aid in cell migration and invasion. In addition, it has been reported that one of the vital functions of PAK4 is to mediate the induction of filopodia in reaction to Cdc42. Thus, considering the inhibitory impact of GL-1196 on cell invasion and PAK4 kinase activity, we detected the influence of GL-1196 on the formation of filopodia. As shown in Figure 7, GL-1196 did inhibit filopodia formation and induce cells elongation in a dose-dependent manner in SGC7901 and BGC823 cells.

### 2.6. GL-1196 Inhibits the Phosphorylation of PAK4 and Its Downstream Signaling Pathways

**Figure 8:**
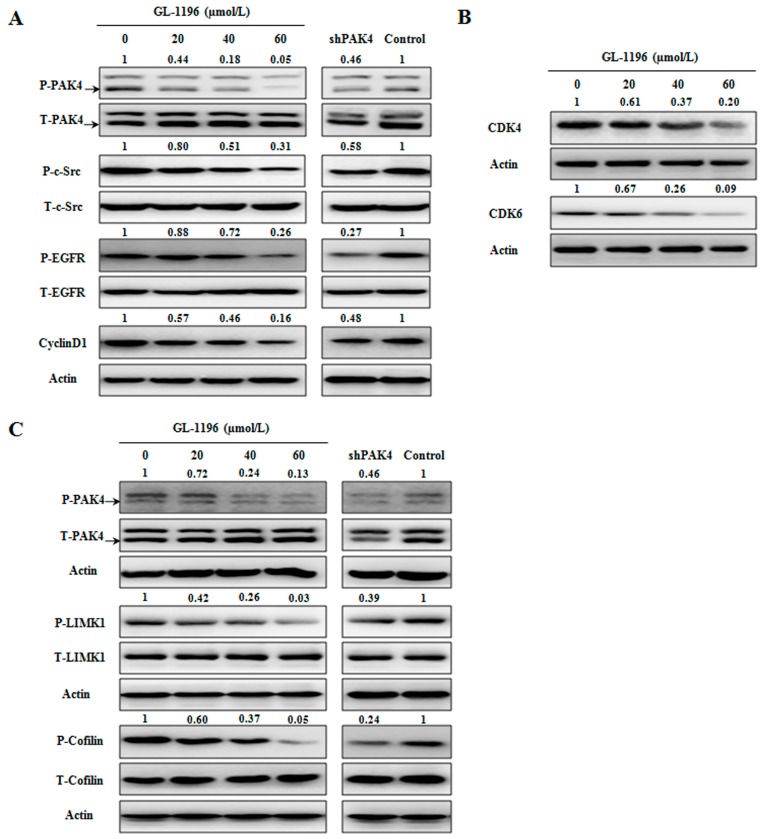
The effect of GL-1196 on PAK4-mediated signaling pathways involving proliferation and invasion. (**A**) GL-1196 inhibits PAK4/c-Src/EGFR/cyclinD1 pathway; (**B**) GL-1196 down-regulated the expression of CDK4/6; (**C**) GL-1196 inhibits PAK4/LIMK1/cofilin pathway.

We have found that GL-1196 could inhibit the proliferation and arrest gastric cancer cells at the G1 phase of cell cycle. Both CyclinD1 and CDK4/6 are key regulators in G1 phase, and it is reported PAK4 could regulate cyclinD1 via PAK4/c-Src/EGFR pathway. Here, we examined the expression levels of these regulators in GL-1196 treated cells. Western blot analysis showed that exposure of SGC7901 to 20/40/60 µmol/L GL-1196 for 24h dramatically decreased the expression levels of phosphorylated PAK4, c-Src, EGFR and cyclinD1, and the effect of GL-1196 treatment was consistent with PAK4 knockdown (Figure 8A). In addition, the expressions of CDK4/6 were also downregulated after GL-1196 exposure (Figure 8B). All of the results above indicated that GL-1196 arrested cells at G1 phase and then suppressed cells growth via down-regulating PAK4/c-Src/EGFR/CyclinD1 pathway and CDK4/6 expression. Next, we explored the mechanism of GL-1196 underlying the inhibition of invasion. Previous studies reported that the PAK4/LIMK1/cofilin signaling pathway had been involved in cell migration. Thus, we investigated the effects of GL-1196 on PAK4/LIMK1/cofilin pathway via western blot analysis. As expected, exposure of SGC7901 cells to various concentrations of GL-1196 decreased the levels of phosphorylated PAK4, LIMK1 and cofilin in a dose-dependent manner (Figure 8C). Moreover, the effect of PAK4/LIMK1/cofilin pathway after exposure to GL-1196 was consistent with that of cells in which PAK4 was knockdown (Figure 8C).

## 3. Discussion

The study presented here described the anti-cancer effect of GL-1196 on human gastric cancer cells for the first time. Our data showed that GL-1196, as a small molecular compound, significantly suppressed the proliferation of gastric cancer cells and arrested cell cycle at G1 phase. Moreover, treatment with GL-1196 resulted in decreased invasive capability and filopodia formation of SGC7901 and BGC823 cells in a dose-dependent manner.

As the most extensively and profoundly studied member among the group II PAKs, PAK4 was considered as a key regulator of the signaling network in cancer cells [23]. It was thought to play roles in numerous cellular processes, including cell survival [22], cell cycle progression [24], and cytoskeletal organization [17]. Given its broad involvement in cellular process, it is well established that PAK4 plays an important role in the tumorigenesis and progression of several kinds of cancers, particularly gastric cancer. Thus, PAK4 has become an attractive potential candidate drug target. Since we have detected that GL-1196 has an inhibitory effect on proliferation and invasion in gastric cancer cells, it is an attractive option for us to explore if GL-1196 could target PAK4 or not. Consistent with our expectation, GL-1196 did markedly inhibit the PAK4 kinase activity in a dose-dependent manner. In addition, the results of docking simulations provided a rationale for the interaction between GL-1196 and PAK4.

Furthermore, in order to elucidate the compound affects cell invasion via targeting PAK4, we performed transwell assays to compare the invasive capability between the cells exposed to GL-1196 and PAK4 knockdown. The results revealed that the inhibitory effect of GL-1196 on cell invasion was indeed due to its impact on PAK4 kinase activity by virtue of the similar effect of GL-1196 treatment and PAK4 knockdown. In addition, we also examined the effect of GL-1196 on filopodia formation that had been previously described as being regulated by PAK4 [17]. The results showed that GL-1196 could indeed inhibit the formation of filopodia and also induce the elongation of cells in SGC7901 and BGC823. Since PAK4 could mediate filopodia formation via direct interaction with active Cdc42 and overexpression of activated PAK4 could induce cell rounding [17,25], it is reasonable that GL-1196 exposure would lead to the reduction of filopodia formation and suppression of cell rounding via inhibiting PAK4 kinase activity.

It is known that CyclinD1 and CDK4/6 are key regulators to mediate the G1 to S phase transition [26,27]. Moreover, previous study revealed that PAK4 could induce the expression of cyclinD1 through PAK4/c-Src/EGFR pathway in ovarian cancer cells. Our observation that GL-1196 arrests the cell cycle at G1 phase is strongly supported by the finding that GL-1196 prominently downregulates PAK4/c-Src/EGFR/cyclinD1 pathway and the expression levels of CDK4/6. Thus, it is reasonable that GL-1196 suppressed the PAK4/c-Src/EGFR/cyclinD1 pathway and CDK4/6 expression, which, in turn, inhibited the transition of cells from G1 to S phase, and finally resulted in the anti-proliferative effect on gastric cancer cells. On the other hand, to clarify the underlying molecular mechanism of inhibitory effect on invasion of GL-1196, we investigated its impact on the PAK4/LIMK1/cofilin signaling pathway that was reported to be essential for cancer cell migration and invasion [25]. Our results showed that GL-1196 effectively suppressed the invasive capability of gastric cancer cells in conjunction with downregulation of PAK4/LIMK1/cofilin pathway, much as previously revealed in prostate cancer cells.

## 4. Materials and Methods

### 4.1. Cell Culture

Human gastric cancer cell lines we used including SGC7901, BGC823, and MKN-45 were cultured in DMEM (dulbecco minimum essential medium) (Life, Waltham, MA, USA) supplemented with 10% fetal calf serum (Life) at 37 °C in a humidified atmosphere of 5% CO_2_ and 95% air.

### 4.2. Reagent

The compound GL-1196 (C_20_H_15_ClN_2_O_3_S, Mass: 398.87) was purchased from Specs (Zoetermeer, The Netherlands) (http://www.specs.net/snpage.php?snpageid=search_show_structure&structureId=AP-853/41701196&guest=Y&message=GUEST%20user%20view...&snmenu=2008111212092858), and the structure of it was shown in Figure 1A. A GL-1196 stock solution (20 mM dissolved in DMSO (Dimethyl Sulphoxide)) was stored at −20 °C then thawed and diluted according to needs in cell culture medium.

### 4.3. MTT Assays

Human gastric cancer cells (1 × 10^4^/well) were seeded in 0.1 mL medium containing 10% FBS (fetal calf serum) in 96-well Corning plates (Tewksbury, MA, USA); 24h later, the cells were treated with the indicated concentrations of GL-1196 for 24 h. Subsequently, 0.01mL MTT (modified tetrazolium salt 3-(4,5-dimethyl-2-thiazolyl)-2,5-diphenyl-2-H-tetrazolium bromide) solution (5 mg/mL in PBS) was added to each well and incubated for 4 h at 37 °C. Then, medium was replaced by 150 μL DMSO. After 15 min of incubation at 37 °C, absorbance at 595 nm was measured using a Microplate Reader (BIO-RAD, Hercules, CA, USA).

### 4.4. Cell-Cycle Analysis by Flow Cytometry

SGC7901 and BGC823 cells were seeded with different concentrations of GL-1196 for 24 h. After incubation, treated cells were then collected, washed with PBS and stained with propidium iodide containing 0.05% RNase. The cells were analyzed using a FACSVantage flow cytometer (BD, Franklin Lakes, NJ, USA) with the CellQuest acquisition and analysis software program (Becton Dickinson and Co., San Jose, CA, USA). Gating was set to exclude cell debris, doublets and clumps.

### 4.5. Lentiviral Production and Infection

We purchased the recombinant lentiviruses involving PAK4-Lentivirus, PAK4-RNAi-Lentivirus and NC-GFP-LV, pGC FU-GFP-LV vectors from Shanghai GeneChem Company (Shanghai, China). In order to stably overexpress PAK4, MKN-45 cells were infected with PAK4-Lentivirus and then selected with puromycin (1.5 µg/mL). The pGC FU-GFP-LV vector was infected into cells as control. Instead, to stably knockdown PAK4, we infected SGC7901 cells using lentiviruses carrying shPAK4 and also selected positive cells by puromycin. The pGC NC-GFP-LV vector was also used as a control.

### 4.6. Cell Invasion Assays

Matrigel invasion assays were performed by chemotaxis chambers with polycarbonate nucleopore membrane. First, the filters (6.5 mm in diameter, 8 μm pore size) should be pre-coated with Matrigel (100 μg/cm^2^) (BD). Then, 1 × 10^5^ cells in 100 μL serum-free DMEM were added to the upper part of each chamber, whereas the lower chambers were filled with 600 μL DMEM medium supplemented with 10% serum. After incubated for 18 h at 37 °C, non-invaded cells were removed from the upper surface of the filter with a cotton swab, and the invaded cells adherent to the lower surface of the filter were fixed, stained and photographed. The number of these cells was counted under high-power magnification.

### 4.7. Real-Time Invasion Monitoring

The invasion assays were performed on CIM-16 plates with 8 μm pore membranes (Roche, Branchburg, NJ, USA). Wells were pre-coated with 20 μL of Matrigel which was allowed to polymerize at 37 °C, 5% CO_2_ for 4 h. Then, the wells of the lower chamber were filled with 160 µL of medium with 10% serum and the top and bottom parts of the plates were assembled together. In addition, the assembled plates were allowed to equilibrate for 2 h at 37 °C, 5% CO_2_ after addition of 50 μL serum-free medium to the top chamber wells. Cells (8 × 10^4^/well) seeded in the top chambers were placed into the xCELLigence system for data collection after incubated for 30 min at room temperature. The xCELLigence software was accurately set to collect impendence data (reported as cell index) at least once an hour.

### 4.8. Immunofluorescence and Confocal Microscopy Analysis

SGC7901 and BGC823 cells grown on glass cover slips were exposed toindicated concentrations of GL-1196 or DMSO for 24 h. Then, cells were fixed in methanol for 15 min, and blocked with normal goat serum for 1 h. Subsequently, cells were incubated with FITC-phalloidin (Sigma, Munich, Germany) for 1 h, washed three times in PBT (PBS with 1% TritonX-100). The DAPI (4′,6-diamidino-2-phenylindole) was used to stain the DNA (blue). Confocal scanning analysis was conducted via a Leika laser confocal scanning microscope (Leika, Wetzlar and Mannheim, Germany). Sequential laser excitation was used to minimize the possibility of fluorescent emission bleed-through.

### 4.9. Kinase Assays

PAK4 kinase assays were performed using the exogenous MBP as substrate to assess activity. For chemical direct effects on PAK4 kinase activity *in vitro*, commercialized PAK4 kinase (Life) was pre-incubated with different concentrations of compound. Kinase reactions were started by adding MBP and a mixture of [γ-^32^P] ATP and 1 mM ATP. Kinase activity was measured in 40 μL of kinase buffer including 10 µCi of [γ-^32^P] ATP (5000 Ci/mmol) for 30 min at 30 °C. Reactions were stopped after mixing with 6× SDS buffer and loading on a 12% SDS-PAGE. Then, proteins were transferred onto PVDF membranes and proteins labeled with ^32^P were visualized by autoradiography with Molecular Imager RX (BIO-RAD). In order to guarantee the equal loading amount, PAK4 were detected by immunoblotting analysis and MBP (Life) was detected by Ponceau stain.

### 4.10. Western Blot Analysis

To investigate the effect of GL-1196 on protein expression, whole cell extracts were prepared from 1 × 10^6^ cells in RIPA lyses buffer (150 mM NaCl, 50 mM Tris/HCl, pH 7.4, 1% Nonidet P-40, 1 mM EDTA, 0.25% Na-deoxycholate and protease inhibitor cocktail). Equal amounts of denatured protein were next separated on SDS-PAGE and transferred to PVDF membranes (Millipore, Darmstadt, Germany). The membrane was blocked in 5% skim milk in TBS-T (137 mM NaCl, 20 mM Tris, pH 7.4, 0.05% Tween-20) for 3 h at room temperature, and the proteins were subsequently incubated with specific antibodies: PAK4, phospho-PAK4 Ser474/PAK5 Ser602/PAK6 Ser560, LIMK1, phospho-LIMK1 Thr508/LIMK2 Thr505, phospho-EGFR Tyr845, cofilin, phospho-cofilin Ser3,c-Src, phosphor-c-Src Tyr416, CDK4, CDK6 (Cell signal, Danvers, MA, USA), cyclinD1 (Neomarker, Waltham, MA, USA), EGFR (Bioworld, Louis Park, MN, USA). All PVDF membranes were detected by chemiluminescence (ECL, Pierce Technology, Waltham, MA, USA). To guaranteethe equal loading amount, membranes were stripped and reprobed with GAPDH antibody (Shang Hai Kangchen, Shanghai, China).

### 4.11. Molecular Modeling

The docking simulation was performed using Glide in Schrödinger version 2014 (TriposAssociates, St. Louis, MO, USA). The non-bond interaction between inhibitor and receptor were displayed in Discovery Studio 4.0 Visual (TriposAssociates).

### 4.12. Statistical Analysis

All statistical analyses were carried out using the SPSS 16.0 software and the results were considered to be significant when the *p* value was <0.05.

## 5. Conclusions 

In summary, our data showed that GL-1196 could suppress the proliferation and invasion of gastric cancer cells via targeting PAK4 and inhibiting PAK4-mediated signaling pathways. Even though further testing of these results *in vivo* is warranted, the present findings do support the viewpoint that GL-1196 may provide a novel therapeutic strategy for advanced metastatic gastric cancer.

## Abbreviations

PAK	p21-activated kinase
CDK	cyclin-dependent kinase
SCG10	superior cervical ganglia 10
LIMK1	LIM domain kinase 1
MBP	myelin basic protein
