# Correction: Zhang et al. GL-1196 Suppresses the Proliferation and Invasion of Gastric Cancer Cells via Targeting PAK4 and Inhibiting PAK4-Mediated Signaling Pathways. *Int. J. Mol. Sci.* 2016, *17*, 470

**DOI:** 10.3390/ijms252413456

**Published:** 2024-12-16

**Authors:** Jian Zhang, Hong-Yan Zhang, Jian Wang, Liang-Hao You, Rui-Zhi Zhou, Dong-Mei Zhao, Mao-Sheng Cheng, Feng Li

**Affiliations:** 1 Department of Cell Biology, Key Laboratory of Cell Biology, Ministry of Public Health and Key Laboratory of Medical Cell Biology, Ministry of Education, China Medical University, Shenyang 110122, China; jiancmu@163.com (J.Z.); zhanghongyan516@163.com (H.-Y.Z.); 18640209060@163.com (L.-H.Y.); zrzym111@126.com (R.-Z.Z.); 2 Key Laboratory of Structure-Based Drug Design & Discovery of Ministry of Education, Shenyang Pharmaceutical University, Shenyang 110016, China; jianwang.chem@gmail.com (J.W.); dongmeiz-67@163.com (D.-M.Z.); mscheng@263.net (M.-S.C.)

In the original publication [1], there was a mistake in Figure 3 as published. In Figure 3B, the image for GL-1196 20 μmol/L was mistakenly inserted due to the similarity of the names. Moreover, according to the Academic Editor’s comments, “SGC 7901” and “BGC 823” in Figure 3A,B have been written as “SGC7901” and “BGC823”; “SGC7901 Cells” and “BGC823 Cells” have been added at the top of the left and right graphs of Figure 3C; and the concentration units “uM” in Figure 3D have been written as “µM”. The corrected Figure 3 appears below. The authors state that the scientific conclusions are unaffected. These corrections were approved by the Academic Editor. The original publication has also been updated.

## Figures and Tables

**Figure 3:**
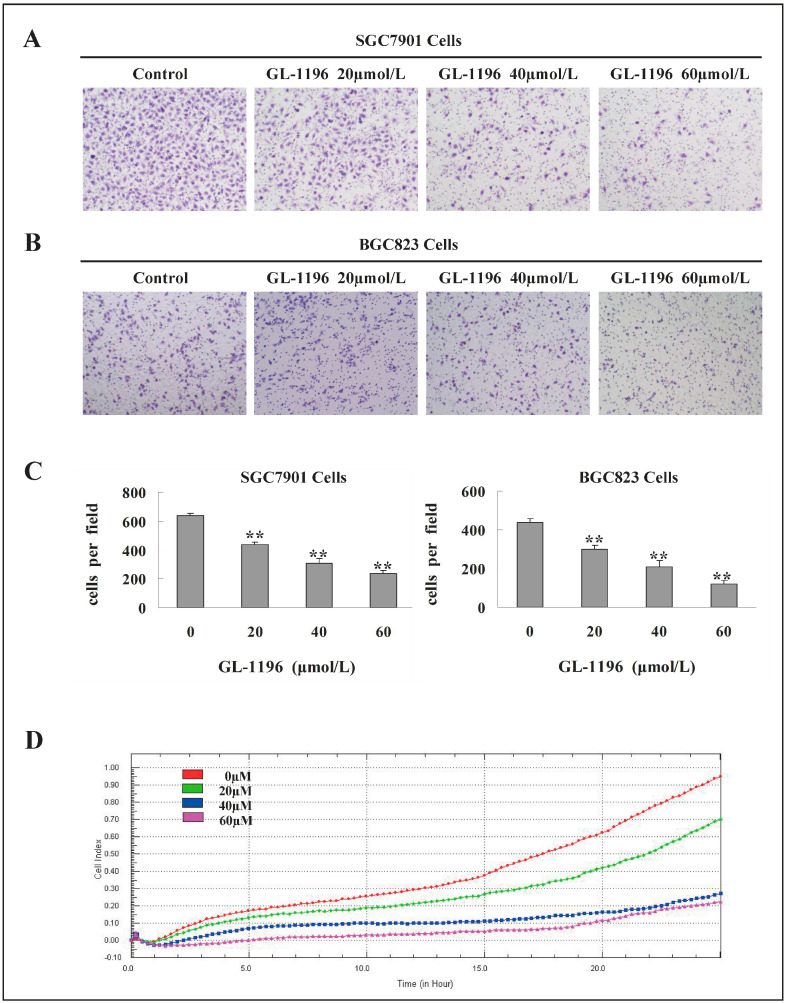
GL-1196 suppresses the invasive capacity of human gastric cancer cells. The invasive capability of SGC7901 (**A**) and BGC823 (**B**) cells was evaluated by chemotaxis chamber matrigel invasion assay. The magnification is 100×, and the number of invading cells is shown as bar diagram ± SEM; (**C**) the left one is for SGC7901 cells; the right one is for BGC823 cells, ** *p* < 0.01; (**D**) the effect of GL-1196 on MKN-45 cells invasive ability was detected by real-time invasion monitoring.
